# Trends in anemia management in US hemodialysis patients 2004–2010

**DOI:** 10.1186/1471-2369-14-264

**Published:** 2013-12-01

**Authors:** Dana C Miskulin, Jing Zhou, Navdeep Tangri, Karen Bandeen-Roche, Courtney Cook, Patti L Ephraim, Deidra C Crews, Julia J Scialla, Stephen M Sozio, Tariq Shafi, Bernard G Jaar, L Ebony Boulware

**Affiliations:** 1 Division of Nephrology, Tufts University School of Medicine, Boston, MA, USA; 2 Division of General Internal Medicine, Johns Hopkins School of Medicine, Baltimore, MD, USA; 3 Division of Nephrology, University of Manitoba, Winnipeg, Manitoba, Canada; 4 Department of Biostatistics, Johns Hopkins Bloomberg School of Public Health, Baltimore, MD, USA; 5 Department of Epidemiology, Johns Hopkins Bloomberg School of Public Health, Baltimore, MD, USA; 6 Welch Center for Prevention, Epidemiology, and Clinical Research, Johns Hopkins Medical Institutions, Baltimore, MD, USA; 7 Division of Nephrology, Johns Hopkins University School of Medicine, Baltimore, MD, USA; 8 Department of Medicine, University of Miami School of Medicine, Miami, FL, USA; 9 Nephrology Center of Maryland, Baltimore, MD, USA; 10 Tufts Medical Center, 800 Washington St., Boston, MA 02111, USA

**Keywords:** Anemia, Erythropoietin stimulating agents, Hemodialysis

## Abstract

**Background:**

There have been major changes in the management of anemia in US hemodialysis patients in recent years. We sought to determine the influence of clinical trial results, safety regulations, and changes in reimbursement policy on practice.

**Methods:**

We examined indicators of anemia management among incident and prevalent hemodialysis patients from a medium-sized dialysis provider over three time periods: (1) 2004 to 2006 (2) 2007 to 2009, and (3) 2010. Trends across the three time periods were compared using generalized estimating equations.

**Results:**

Prior to 2007, the median proportion of patients with monthly hemoglobin >12 g/dL for patients on dialysis 0 to 3, 4 to 6 and 7 to 18 months, respectively, was 42%, 55% and 46% declined to 41%, 54%, and 40% after 2007, and declined more sharply in 2010 to 34%, 41%, and 30%. Median weekly Epoeitin alpha doses over the same periods were 18,000, 12,400, and 9,100 units before 2007; remained relatively unchanged from 2007 to 2009; and decreased sharply in the patients 3–6 and 6–18 months on dialysis to 10,200 and 7,800 units, respectively in 2010. Iron doses, serum ferritin, and transferrin saturation levels increased over time with more pronounced increases in 2010.

**Conclusion:**

Modest changes in anemia management occurred between 2007 and 2009, followed by more dramatic changes in 2010. Studies are needed to examine the effects of declining erythropoietin use and hemoglobin levels and increasing intravenous iron use on quality of life, transplantation rates, infection rates and survival.

## Background

Efforts to identify an anemia management strategy that optimizes clinical outcomes and quality of life among hemodialysis (HD) patients have received substantial attention in recent years [1,2]. Until recently, ESA use in patients with chronic kidney disease (CKD) had been steadily increasing [3]. Average doses of erythropoiesis stimulating agents (ESAs) in 2006 were threefold higher than they were in 1991 and ESAs accounted for $2 billion of Medicare expenditures [3]. Such changes occurred despite early experiences indicating that there was an increased risk of thromboembolic events, worsened hypertension, and seizures with treatment with ESAs. In 2006, a major trial found an increased risk of cardiovascular events and death of treating CKD patients to higher versus lower Hb targets [4]. Two other trials, one involving HD patients and published in 1998 [5], and the other, involving CKD patients and also published in late 2006 [6], reported no benefit with treating to higher Hb values.

In response to these findings and studies showing an increase in cancer deaths in oncology patients, the U.S. Food and Drug Administration (FDA) issued a black box warning on ESA labels in March 2007, recommending ESAs be used at the lowest possible dose to avoid transfusion [7]. At this same time, a short term, randomized trial found that treatment with 1 g of intravenous (IV) iron in patients with serum ferritin 500–1000 and TSat <25% led to a significant increase in Hb and decline in EPO dose [8]. In late 2009, a fourth major of targeting a high vs. lower Hb was published, and showed an increase in stroke (a secondary endpoint) with targeting higher Hb values in CKD patients [9]. The FDA issued new safety regulations, requiring that ESAs be prescribed under a risk evaluation and mitigation program [10].

Recent changes to reimbursement for dialysis care may have also influenced practice. Prior to January 1, 2011, the Centers for Medicare and Medicaid Services (CMS) reimbursed dialysis facilities for ESAs at the average wholesale price plus 6 percent [11]. In August 2010, CMS announced the final rules for the ESRD Prospective Payment System (ESRD PPS) or bundled payment system, under which injectable medications administered in HD units, including ESAs, each be reimbursed at a fixed rate, regardless of dose, beginning January 1, 2011 [11].

We sought to determine the potential influence of clinical trial results and changes in safety regulations with changes in reimbursement policies on anemia management practices in a representative U.S. HD population.

## Methods

### Study population

We performed a series of cross-sectional analyses to characterize patterns of anemia management during the first 18 months of HD from 2004 to 2010 in Dialysis Clinic, Inc (DCI), a medium-sized not-for-profit US dialysis provider with approximately 200 dialysis units across the US. We included patients who survived at least three months from the time of dialysis initiation, had at least one HD treatment and at least one hemoglobin (Hb) measurement. We excluded patients who received peritoneal dialysis or home HD between 2004 and 2010.

### Data

We obtained data from DCI’s electronic medical information system. Laboratory measures for Hb, serum ferritin, iron and transferrin had been processed at the DCI Central Laboratory (Nashville, TN), which is certified by the Clinical Laboratory Improvement Amendments. We calculated TSat using the formula: (TSat = iron/transferrin*70.9). If more than one value of a laboratory parameter (e.g., Hb) existed in a month, we took the median of all values. The cumulative IV iron dose per month was calculated as the sum of iron dextran, iron sucrose or iron gluconate administered each month. If there was no record of IV iron administered for a treatment, the dose was zero. The median weekly EPO dose per month was calculated as the cumulative weekly EPO dose first, and then we took the median of the weekly doses over the month. The study was conducted in accordance with the Declaration of Helsinki and was approved by the Johns Hopkins Medicine Institutional Review Board (Baltimore, Maryland).

### Statistical analysis

We stratified analyses according to patients’ time on HD (i.e. 0–3, 4–6 and 7–18 months after starting HD) to account for differences in ESA and iron dosing at dialysis initiation when patients often require more ESA and iron than later on.

We examined trends in anemia management over three time periods: 1) 2004–2006; 2) 2007–2009, which follows the publication of a major trial showing harm with targeting a higher Hb in CKD patients and another showing no benefit [4,6] as well as the FDA’s issuance of a black box warning on the EPO label advising against targeting higher Hb levels [7]; and 3) 2010, which follows publication of another trial showing increase in strokes with treating CKD patients to a higher Hb target [9] and release of the Final Rule for a bundled CMS payment system [11] to be enacted January 1, 2011. To examine whether the changes in anemia management between time periods were statistically significant, we modeled the anemia parameters for each individual using generalized estimating equations (GEE) models which account for ‘within subject’ as well as ‘between clinic’ correlations. Hb >12 g/dL and Hb <10 g/dL were modeled as binary outcomes, and ferritin, TSat, and EPO dose were modeled as continuous outcomes. To examine the trends in IV iron doses overtime, we used two-stage GEE models as a substantial proportion of patients did not receive IV iron in a given month. In the first stage, we modeled the binary outcome of “any” versus “zero” doses; and for the second stage we modeled iron dose as a continuous variable, among those receiving iron. We created linear spline terms with knots at January 2007 and January 2010 to test for differences in the initial values (i.e. intercepts) and rates of change (i.e. slopes) during the three time periods (2004–2006, 2007–2009 and 2010). We adjusted all models for age, race/ethnicity, gender, and diabetes status. Analyses were conducted using SAS 9.2 (Cary, NC).

## Results

### Study population

We observed anemia management practices among 12,281, 13,288, and 6,142 patients who dialyzed between 2004–2006, 2007–2009 and 2010, respectively, at dialysis units within Dialysis Clinic Inc, (DCI). Patient characteristics across the three time periods were similar to those of patients initiating dialysis across the US, with the exception of a higher proportion of African American (36% vs. 28%) and a lower proportion of Hispanics (6% vs. 14%) [3]. The proportion of the study population that was African American or Hispanic, 45–64 years old, and with ‘other’ causes of ESRD, increased over the three time periods (Table 1).

**Table 1:** Patient characteristics, by time period

			**Years**		
		**2004–2006**	**2007–2009**	**2010**	**p-value**^**1**^
Total no. (N)		12,281	13,288	6,142	<0.01
Age at first dialysis treatment at DCI, mean (SD)		62.2 (15.4)	62.0 (15.3)	61.9 (15.3)	0.35
Age category at first dialysis treatment at DCI, N (%)	18–44	1,773 (14.4)	1,882 (14.2)	864 (14.1)	<0.01
	45–64	4,736 (38.6)	5,374 (40.4)	2,546 (41.5)	
	≥65	5,772 (47.0)	6,032 (45.4)	2,732 (44.5)	
Sex, N (%)	Male	6,854 (55.8)	7,439 (56.0)	3,452 (56.2)	0.88
	Female	5,427 (44.2)	5,849 (44.0)	2,690 (43.8)	
Race, N (%)	African American	4,357 (35.5)	4,766 (35.9)	2,264 (36.9)	<0.01
	White	7,050 (57.4)	7,550 (56.8)	3,425 (55.8)	
	Other^2^	802 (6.5)	867 (6.5)	377 (6.1)	
	Unknown	72 (0.6)	105 (0.8)	76 (1.2)	
Ethnicity, N (%)	Hispanic	682 (5.6)	828 (6.2)	421 (6.9)	<0.01
	Non-Hispanic	11,535 (93.9)	12,308 (92.6)	5,598 (91.1)	
	Unknown	64 (0.5)	152 (1.1)	123 (2.0)	
Primary cause of ESRD, N (%)	Diabetes	5,529 (45.0)	6,001 (45.2)	2,771 (45.1)	0.03
	Hypertension	3,511 (28.6)	3,847 (29.0)	1,758(28.6)	
	Glomerulonephritis	1,152 (9.4)	1,121 (8.4)	526 (8.6)	
	Other	2,045 (16.7)	2,284 (17.2)	1,078 (17.6)	
	Unknown	44 (0.4)	35 (0.3)	9 (0.2)	

^1^Chi-square test.

^2^American Indian, Asian or Pacific Islander.

### Unadjusted trends in anemia management

Per Table 2 we find that the percent of patients with Hb >12 g/dL decreased between 2004–2006 and 2007–2009 and fell even more sharply in 2010. This was accompanied by an increase in Hb <10 g/dL and a parallel decrease in the percent of patients with a Hb >12 g/dL. Median Epoetin Alfa (EPO) doses were similar over 2004 to 2006 and 2007–2009 but decreased dramatically in 2010. The percentage of patients receiving monthly intravenous (IV) iron also was lower in 2010, compared to the periods before 2007. However, among patients who received IV iron, the median doses were higher between 2007 to 2009 and in 2010 compared to before 2007. Median values of serum ferritin, and transferrin saturation (TSat) also increased across periods.

**Table 2:** Anemia management parameters by time on dialysis and dialysis incidence year

	**Time on dialysis**		**Incidence year**	
		**2004–2006**	**2007–2009**	**2010**
Hemoglobin (g/dl), Median (Q25, Q75)	0–3 month	11.7 (10.4, 12.9)	11.6 (10.3, 12.8)	11.4 (10.0, 12.5)
	3–6 month	12.3 (11.3, 13.2)	12.3 (11.4, 13.0)	12.0 (11.2, 12.7)
	6–18 month	12.1 (11.2, 12.9)	11.9 (11.1, 12.7)	11.8 (10.9, 12.5)
Percent with Hemoglobin <10 (monthly), Median (Q25, Q75)	0–3 month	12.8 (8.2, 30.4)	13.3 (7.6, 34.0)	17.1 (9.3, 42.8)
	3–6 month	7.0 (6.6, 7.1)	6.7 (6.5, 7.4)	7.7 (6.6, 8.0)
	6–18 month	6.2 (6.0, 6.5)	6.8 (6.5, 6.9)	8.9 (8.3, 9.3)
Percent with Hemoglobin > 12 (monthly), Median (Q25, Q75)	0–3 month	42.0 (17.8, 57.1)	40.6 (14.5, 57.4)	33.6 (9.08, 49.5)
	3–6 month	54.9 (50.5, 58.7)	54.2 (49.8, 58.2)	41.2 (35.8, 48.4)
	6–18 month	45.9 (44.6, 49.7)	39.9 (36.9, 46.1)	29.5 (25.7, 35.9)
Weekly ESA doses (Unit), Median (Q25, Q75)	0–3 month	18000 (9000, 30000)	20000 (9000, 30600)	18000 (8000, 30000)
	3–6 month	12400 (4800, 24000)	13500 (4800, 25200)	10200 (2600, 21000)
	6–18 month	9100 (3900, 19500)	9600 (3900, 20100)	7800 (2400, 16800)
Percent of patients receiving monthly IV iron, Median (Q25, Q75)	0–3 month	71.1 (62.5,72.5)	77.8 (67.9,78.5)	76.0 (72.6,77.4)
	3–6 month	71.0 (69.7,72.0)	74.5 (73.6,76.2)	70.3 (68.9,73.6)
	6–18 month	65.7 (64.5,68.2)	67.4 (65.9,70.4)	60.6 (58.1,62.2)
Monthly iron doses (mg) for those who had received iron, Median (Q25, Q75)	0–3 month	400 (200,800)	475 (200,800)	500 (300,850)
	3–6 month	300 (200,500)	300 (200,500)	400 (200,600)
	6–18 month	200 (125,400)	225 (125,400)	300 (200,500)
TSat(%), Median (Q25, Q75)	0–3 month	18.4 (13.7, 24.8)	18.3 (13.9, 24.5)	21.5 (16.1, 28.4)
	3–6 month	22.6 (17.0, 30.3)	22.6 (17.2, 30.0)	24.4 (19.1, 32.8)
	6–18 month	23.6 (18.3, 30.5)	23.6 (18.4, 30.5)	25.6 (20.3, 33.3)
Ferritin (ng/ml), Median (Q25, Q75)	0–3 month	284 (136, 533)	312 (157, 566)	354 (179, 624)
	3–6 month	447 (243, 687)	499 (287, 744)	600 (360, 858)
	6–18 month	592 (376, 796)	654 (436, 893)	707 (474, 996)

### Time trends in anemia management

#### *Hemoglobin*

**Figure 1:**
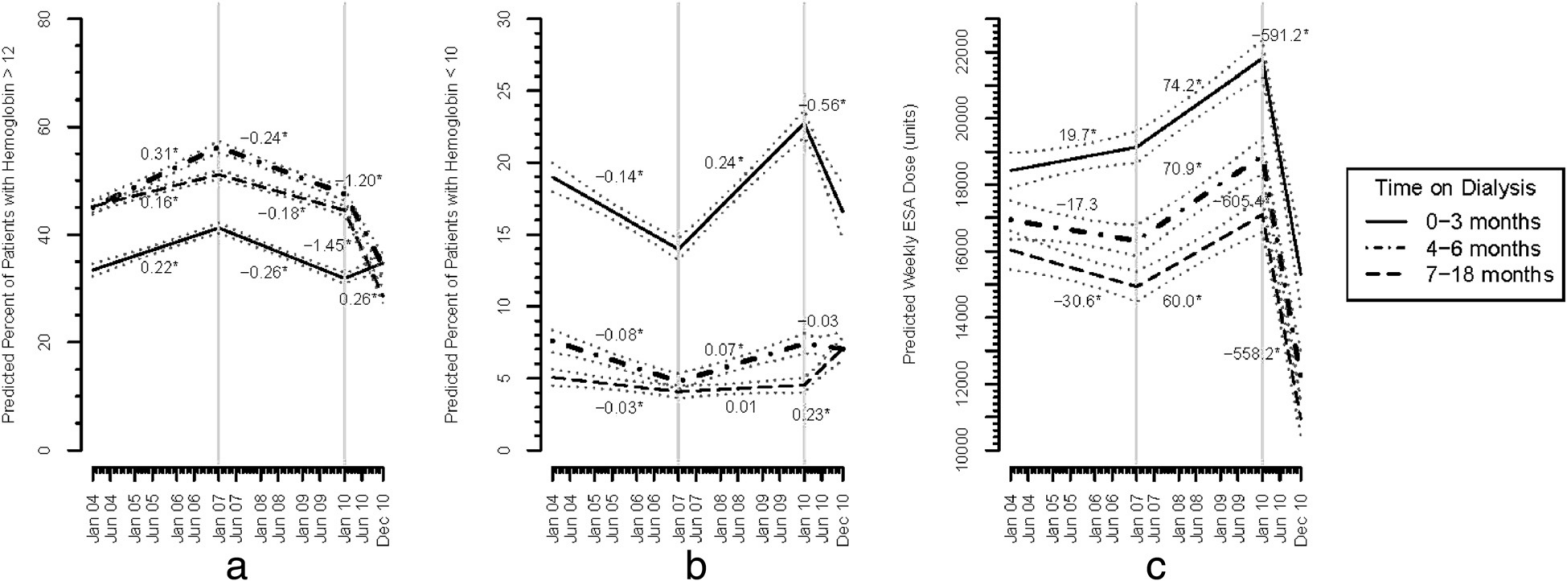
**Multivariable adjusted Hb and EPO doses 2004-2010 among patients 0–3, 4–6 and 7–18 months on dialysis.** Panel **(a)** Percentages of patients with Hb < 10 g/dL; Panel **(b)** Percentages of patients with Hb > 12 g/dL; Panel **(c) **Weekly EPO doses. Vales are based on white males greater than 65 years old with diabetes as the cause of ESRD. The slopes represent monthly rates of change during each of the three time periods (2004–2006, 2007–2009 and 2010). The “*” indicate that the slope is significantly different from 0. The grey dotted lines are 95% confidence intervals for the adjusted values. The difference in slopes for Hb < 10, Hb >10 g/dL and EPO dose within each ‘time on dialysis’ subgroup between 2004–2006 and 2007–2009 and between 2007–2009 and 2010 were statistically significant except for Hemoglobin < 10 g/dL in the 4–6 month patient subgroup between 2007–2009 and 2010.

Results are described separately by subgroups defined by the time since starting dialysis (0–3, 4–6, 7–18 months), as trends were different depending on dialysis vintage. Between January 2004 and December 2006, the percentage of patients with Hb >12 g/dL increased among patients on dialysis for 0–3, 4–6, and 7–18 months, from 33 to 41%, 45 to 56%, 45 to 51%, respectively, but declined between 2007–2009 and by the end of 2009, were at values seen back in 2004. The proportion with Hb >12 g/dL fell more sharply in 2010 except in new patients 0–3 months on dialysis where there was an increase in Hb > 12 g/dL (Figure 1a).

The changes in the frequency of Hb <10 g/dL (Figure 1b) were the opposite of the trends described above for Hb >12 g/dL, with a decrease in Hb <10 g/dL between 2004–2006, an increase between 2007–2009 and further increases in 2010, such that by December, 2010, 17% of patients who had been receiving dialysis for 0–3 months, 5% of patients dialyzed for 4 to 6, months and 7% of patients dialyzed for 7–18 months had a Hb <10 g/dL. Changes in the frequency of Hb > 12 g/dL and Hb <10 g/dL across the 3 periods were statistically significant for all comparisons except for the change in Hb <10 g/dL between 2007–2009 and 2010 in the subgroup of patients who had been dialyzing for 4–6 months.

#### *Epoetin alfa utilization*

Weekly EPO dose was essentially unchanged between 2004 and 2006 (Figure 1c). Between 2007–2009, EPO dose increased from 19,133 to 21,805 units among patients dialyzing for 0–3 months, 16,317 to 18,870 units among patients dialyzing 4–6 months and 14,931 to 17,091 units among patients dialyzing 7–18 months. Doses declined sharply in 2010 to 15,302, 12,211 and 10,951 units in these same groups, respectively, which represents a 30%, 35% and 36% decline. The rates of change in EPO dose across the three time periods were statistically significant in each subgroup.

#### *Intravenous iron therapy*

Between January 2004 and December 2006, the percentage of patients receiving monthly IV iron, increased from 70% to 74%, 70% to 74%, and 69% to 71% among patients who had been treated with HD for 0–3, 4–6 and 7–18 months, respectively. There were small increases in each of the three subgroups between 2007–2009 and a small decline in 2010.

**Figure 2:**
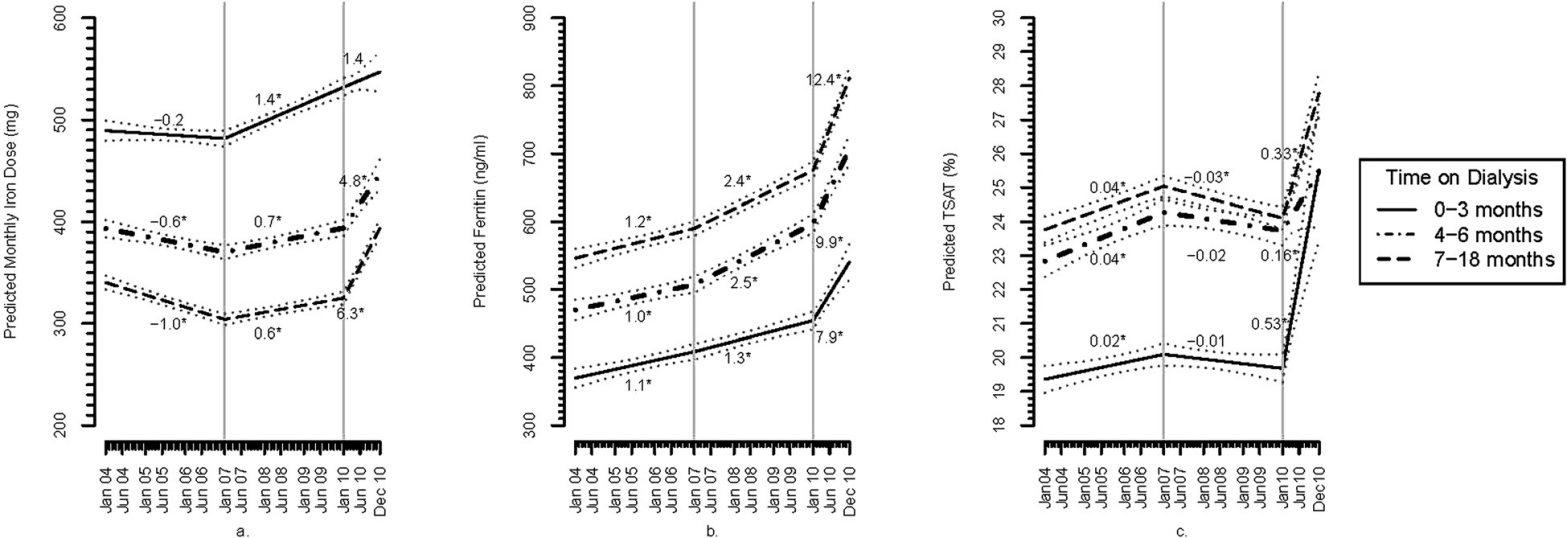
**Multivariable adjusted iron doses and markers of iron stores 2004-2010 among patients 0–3, 4–6, and 7–18 months on dialysis.** Panel **(a)** Monthly iron dose (mg); Panel **(b)** Serum ferritin (ng/ml); Panel **(c)** TSat (%). Values are based on white males greater than 65 years old with diabetes as the cause of ESRD. The slopes represent monthly rates of change for each of the three time periods (2004–2006, 2007–2009 and 2010). The “*” indicate that the slope is significantly different from 0. The grey dotted lines are 95% confidence intervals for the predicted values. The difference in slopes between 2004–2006 and 2007–2009 and between 2007–2009 and 2010 are statistically significant within each ‘time on dialysis’ subgroup for each of the parameters except for the comparisons of monthly iron dose in the 0–3 month subgroup between 2007–2009 and 2010, and ferritin in the 0–3 month subgroup between 2004–2006 and 2007–2009.

Per Figure 2a, among patients receiving iron, there was no clinically meaningful change in monthly IV iron doses between 2004 to 2006 or between 2007 to 2009. Between January 2010 and December 2010, the adjusted monthly IV iron dose increased from 532 to 547, 394 to 446 and 325 to 394 mg respectively, among patients 0–3, 4–6 and 7–18 months on dialysis. The increase in iron dose before and after 2010 was statistically significant in the prevalent subgroups (dialyzing 4–6 and 7–18 months).

#### *Transferrin saturation and ferritin*

There was no significant change in multivariable adjusted TSat values between 2004–2006 and 2007–2009 for any subgroup by time on dialysis (Figure 2b). Between January and December 2010, there were statistically significant increases in TSat values from 20% to 26%, 24% to 25% and 24% to 28% in patients 0–3, 4–6, and 7–18 months on dialysis.

Serum ferritin steadily increased from 2004 through the end of 2010 in each of the subgroups (Figure 2c), with changes of 370 to 541 mg, 470 to 707, and 546 812 ng/ml, in patients who had received dialysis for 0–3, 4–6 and 7–18 months, respectively. The rates of change in TSat and serum ferritin levels across the 3 time periods were statistically significant for all subgroups except for ferritin in the incident patients (dialyzing 0–3 months) between 2004-2006 and 2007- 2009.

## Discussion

Our results suggest that the 2006 publication of a clinical trial demonstrating harm with targeting higher Hb levels [4] and the FDA’s issuance of a black box warning on the epoeitin label [7] led to relatively modest changes in anemia management as compared with the much sharper declines in Hb and EPO doses that were seen in 2010 after publication of another trial showing harm with higher Hb values [9] and CMS’s release of the final rule for a revised payment system [11]. Over the course of 2010, there was a nearly 30% decline in the proportion of patients with Hb greater than12 g/dL and a more than 30% decline in median EPO doses.

Our observations are consistent with the USRDS Annual Data Report [12], which showed more dramatic reductions in EPO doses and in the proportion of patients with Hb levels greater than 12 g/dL in 2010 than in earlier years. It is impossible to tell whether these changes in anemia management practices were influenced more by the publication of a second major trial showing harm with targeting a higher Hb value or preparations for bundled payment policies for dialysis care, as the two occurred at the same time. Practice may not have changed following publication of a major trial showing harm with targeting high Hb in late 2006 because it was conducted in CKD patients and nephrologists may have considered that these results did not apply to HD patients. However, if this was the explanation, the TREAT trial, published in late 2009, should also not have led to the large changes in practice seen in 2010, as it was also conducted in CKD patients. These findings may suggest that US dialysis units may be much more sensitive to changes in payment policies than research findings and FDA regulations. We speculate that the changes in payment policies likely reinforced and incentivized clinicians’ further modifications to their practice patterns to achieve still lower hemoglobin levels through 2010, primarily through more sparing use of EPO. The immediate and dramatic declines in EPO use observed in our study reflect the high costs of EPO, as well as dialysis organizations’ motivation and ability to minimize use of costly ESAs while achieving appropriate anemia management goals through centralized prescribing protocols [13].

Only a small proportion of patients had Hb values less than 10 mg/dl in 2010, though these data predate changes in the CMS Quality Incentive Program (QIP), under which the penalty for Hb values falling below 10 g/dL was removed in 2013 [14]. Per data from the US Dialysis Outcomes and Practice Patterns Study (DOPPS) EPO doses have continued to decline through 2011, as has the prevalence of Hb less than 10 g/dL [15]. An increase in blood transfusions, longer transplant waiting times as a result of sensitization, as well as declines in physical functioning, cognition and health-related quality of life are potential consequences of a decline in Hb values in the HD population. It is clear that some patients would be willing to accept higher risks of harms (e.g., stroke, myocardial infarction or death) in return for improved cognitive and physical functioning. To individualize anemia management, we need sensitive measures of cognition, physical health and quality of life as well as open discussion among physicians and their patients about their values and preferences. Attention toward patients’ preferences regarding the potential tradeoffs associated with anemia management strategies may be particularly pertinent in light of the downward shift in distribution of Hb levels that we observed and we anticipate will continue, with the removal of the QIP penalty for Hb less than 10 g/dL in 2013 [14].

Our study is strengthened by its examination of several years of anemia management in a large, representative US dialysis population. We are unaware of other studies explicitly exploring the influence of secular events on trends in anemia management. Our study also has limitations. DCI is a not-for-profit dialysis provider, which may respond differently to secular factors than for-profit dialysis providers. We suspect this is not the case, however, as the general trends reported in this study are similar to those reported by the USRDS [16]. We do not examine trends in practice beyond 18 months after starting dialysis, as at the time of data extraction, the number of patients in recent years with more prolonged follow-up was limited. It is possible that the trends seen here are not applicable to very prevalent patients (greater than 18 months after starting dialysis). Although we attempted to describe trends in anemia management in relation to secular events, we cannot make causal inferences, as clinical trial publications overlapped with changes in product label regulations and in reimbursement. Nonetheless, we believe our detailed description of changes in several key indices of anemia management in relation to the publication of major clinical trial results, and changes in regulatory and reimbursement policies, provide insight into numerous potential influences that affect anemia management and patient outcomes.

## Conclusion

In conclusion, modest changes in anemia management occurred after 2007, with more substantial changes after 2010. Both scientific evidence indicating harms with targeting higher Hb levels, and changes in dialysis reimbursement policies appear to have been associated with these changes, though more dramatic changes coincided with the latter. Study of the effects of treating to lower Hb values, and the use of less ESA and more IV iron, on morbidity, mortality, transplant waiting times, and health related quality of life are needed.

## Competing interests

The authors declare that they have no competing interests.

## Authors’ contributions

DM, NT, LEB, DC, contributed to conception and design, interpretation and drafting the manuscript and gave final approval for submission to be published. DC, JS, SS, TS and BJ contributed to conception and design, interpretation of results and edited the manuscript for important intellectual content. PE acquired the data and contributed to design and interpretation of results. JZ, CC and KB-R contributed to design, and conducted analyses, assisted with interpretation and to writing and editing the draft. All authors read and approved the final manuscript.

## Pre-publication history

The pre-publication history for this paper can be accessed here:

http://www.biomedcentral.com/1471-2369/14/264/prepub
